# Sulindac sulfide as a non-immune suppressive γ-secretase modulator to target triple-negative breast cancer

**DOI:** 10.3389/fimmu.2023.1244159

**Published:** 2023-10-13

**Authors:** Fokhrul Hossain, Deniz A. Ucar, Giulia Monticone, Yong Ran, Samarpan Majumder, Kristina Larter, Hanh Luu, Dorota Wyczechowska, Soroor Heidari, Keli Xu, Sudarvili Shanthalingam, Margarite Matossian, Yaguang Xi, Matthew Burow, Bridgette Collins-Burow, Luis Del Valle, Chindo Hicks, Jovanny Zabaleta, Todd Golde, Barbara Osborne, Lucio Miele

**Affiliations:** ^1^Department of Genetics, Louisiana State University Health Sciences Center, New Orleans (LSUHSC-NO), New Orleans, LA, United States; ^2^Department of Pharmacological and Chemical Biology, Emory University, Atlanta, GA, United States; ^3^Department of Interdisciplinary Oncology, LSUHSC-NO, New Orleans, LA, United States; ^4^Department of Neurobiology and Anatomical Sciences, University of Mississippi Medical Center, Jackson, MS, United States; ^5^Department of Veterinary and Animal Sciences, University of Massachusetts, Amherst, MA, United States; ^6^School of Medicine, Tulane University, New Orleans, LA, United States; ^7^Department of Pathology, Louisiana State University Health Sciences Center - New Orleans (LSUHSC-NO), New Orleans, LA, United States

**Keywords:** triple-negative breast cancer, sulindac sulfide, immunotherapy, Notch, T-cells

## Abstract

**Introduction:**

Triple-negative breast cancer (TNBC) comprises a heterogeneous group of clinically aggressive tumors with high risk of recurrence and metastasis. Current pharmacological treatment options remain largely limited to chemotherapy. Despite promising results, the efficacy of immunotherapy and chemo-immunotherapy in TNBC remains limited. There is strong evidence supporting the involvement of Notch signaling in TNBC progression. Expression of Notch1 and its ligand Jagged1 correlate with poor prognosis. Notch inhibitors, including g-secretase inhibitors (GSIs), are quite effective in preclinical models of TNBC. However, the success of GSIs in clinical trials has been limited by their intestinal toxicity and potential for adverse immunological effects, since Notch plays key roles in T-cell activation, including CD8 T-cells in tumors. Our overarching goal is to replace GSIs with agents that lack their systemic toxicity and ideally, do not affect tumor immunity. We identified sulindac sulfide (SS), the active metabolite of FDA-approved NSAID sulindac, as a potential candidate to replace GSIs.

**Methods:**

We investigated the pharmacological and immunotherapeutic properties of SS in TNBC models *in vitro*, *ex-vivo* and *in vivo*.

**Results:**

We confirmed that SS, a known γ-secretase modulator (GSM), inhibits Notch1 cleavage in TNBC cells. SS significantly inhibited mammosphere growth in all human and murine TNBC models tested. In a transplantable mouse TNBC tumor model (C0321), SS had remarkable single-agent anti-tumor activity and eliminated Notch1 protein expression in tumors. Importantly, SS did not inhibit Notch cleavage in T- cells, and the anti-tumor effects of SS were significantly enhanced when combined with a-PD1 immunotherapy in our TNBC organoids and *in vivo*.

**Discussion:**

Our data support further investigation of SS for the treatment of TNBC, in conjunction with chemo- or -chemo-immunotherapy. Repurposing an FDA-approved, safe agent for the treatment of TNBC may be a cost-effective, rapidly deployable therapeutic option for a patient population in need of more effective therapies.

## Introduction

Triple-negative breast cancer (TNBC) is a heterogeneous group of clinically aggressive breast cancers that accounts for approximately 10-15% of all breast cancer cases (1). TNBCs are pathologically negative for estrogen receptor (ER-), progesterone receptor (PR-), and human epidermal growth factor receptor 2 (HER2) amplification, which limits the use of targeted therapies (2–4). TNBC patients have a high mortality rate due to metastatic or locally recurrent disease, chemo- and radio-resistance (5–8). Molecular heterogeneity among TNBC patients, intra-tumoral clonal and phenotypic heterogeneity, cancer stem-like cells (CSCs) as well as tumor microenvironment plasticity make TNBC a major clinical challenge (9–12). Numerous studies suggest the involvement of Notch signaling in TNBC (13–20). Expression of intra-tumoral Notch1 mRNA and protein correlates with poor prognosis and survival in TNBC (21–23). Approximately 13% of TNBC contain gain-of-function mutations in NOTCH1, 2 or 3 that predict sensitivity to γ-secretase inhibitors (GSIs) (24, 25). Numerous groups have investigated the targeting of Notch signaling in breast cancer (26–29). Evidence shows that TNBC CSCs emerging after chemotherapy or treatment with targeted agents are often Notch-dependent (30–38). Notch inhibitors, e.g. GSIs, are quite effective in preclinical models of TNBC, where they eliminate CSC resistance to chemotherapy (32, 33, 35, 39–42). However, GSIs have had minimal success in TNBC clinical trials due to their intestinal toxicity, limited effectiveness as monotherapy, and adverse effects on immune cells (43–46). Notch signaling is required for T-cell activation, including CD8 effector T-cells that participate in anti-tumor responses (47–49). To overcome this impasse, we sought non-immune suppressive, FDA-approved agents with γ-secretase inhibitor/modulator activities. We focused on sulindac sulfide (SS), the active metabolite of the nonsteroidal anti-inflammatory drug (NSAID) sulindac, which is FDA-approved and has γ-secretase modulator (GSM) activity (50).

NSAIDs are potent anti-inflammatory agents that inhibit cyclooxygenase (COX) enzymatic activity and are used to treat inflammatory conditions and chronic pain (51). COX-1 and -2 generate prostaglandins and thromboxanes from arachidonic acid. These eicosanoids play major roles in inflammation and other physiological processes, including renal function, clot formation, and gastrointestinal protection (52–55). In addition to their uses as anti-inflammatory agents, NSAIDs have been studied in the context of cancer prevention. Numerous epidemiologic and experimental studies have shown that NSAIDs have chemopreventive activity against several cancer types including breast and colorectal cancer (56–61). Harris et al. reported that long-term regular use of any NSAID reduced the risk of breast cancer by 28% (62). A prospective cohort study of early-stage breast cancer survivors suggested that regular use of NSAIDs was associated with a significantly decreased risk of breast cancer recurrence (63). Sulindac, a NSAID prodrug, is metabolized by liver enzymes and colonic bacteria to sulindac sulfide (SS) and sulindac sulfone (SF) (64). SS, but not SF, inhibits COX-1 and COX-2 enzymes, suppressing prostaglandin synthesis (64, 65). COX-2 is highly expressed in TNBC, and its expression is correlated with poor survival in basal-like TNBC (66). SS has been investigated as a therapeutic agent for many cancers including breast and colon (67–71). However, the mechanisms of the antitumor activity of SS remain unclear. Through its primary targets COX-1 and COX-2, SS prevents the production of prostaglandin E_2_ (PGE_2_), a highly immune-suppressive inflammatory mediator that is well-known to dampen T-cell responses, including CD8 T-cell activity (72–74). PGE_2_, produced by tumor cells, tumor-associated macrophages (TAMs), and regulatory T-cells (Tregs), has multiple immune-modulatory effects in the tumor microenvironment, leading to decreased antitumor dendritic cell (DC) and Th1 T-cell functions and increased pro-tumor Treg, myeloid derived suppressor cell (MDSC) and M2 TAM functions (75–79). Breast cancer patients express high levels of PGE_2_, which are inversely associated with patients’ prognosis and positively correlated with the clinically aggressiveness of breast cancer (80, 81). In breast CSCs, PGE_2_ induces Notch and Wnt activity through the PGE_2_ receptor EP4 and the PI3K-AKT-GSK3β cascade (72, 82, 83). Importantly, off-target effects may contribute to the activity of SS. SS is reported to inhibit IKKα and β phosphorylation and NF-κB activity (41, 84, 85), including in TNBC cells (86). We and others have previously reported that Notch1 activates IKKα and NF-κB in TNBC (14) and T-ALL (87, 88). NF-κB induces Notch ligands Jagged1 and 2, which are key to the immune-suppressive activity of MDSCs (14). Jagged1-Notch signaling in TNBC modulates the immune microenvironment by promoting the recruitment of TAMs, the production of TGF-β, and TAM maturation (89).

γ-Secretase modulators (GSMs) do not competitively inhibit γ-secretase, but modify its catalytic activity by indirectly altering enzyme-substrate complexes (90). The Golde laboratory and others have shown that some NSAIDs including sulindac have GSM activity (90–92). Therefore, we investigated whether SS may modulate Notch signaling in TNBC.

We found that SS decreases the growth of mammospheres in a Notch-dependent fashion. SS was active in mammospheres from human and mouse TNBC models including two different patient-derived xenografts (PDXs). *In vivo*, SS had single-agent anti-tumor activity in a Notch-driven TNBC model without causing diarrhea or immune suppression, and increased the efficacy of anti-PD1 (α-PD1 henceforth) checkpoint inhibitor treatment. Our data indicate that SS repurposing may be an attractive strategy to inhibit Notch and simultaneously promote tumor immunity in TNBC.

## Materials and methods

### Cell lines

All cancer cell lines were cultured in DMEM medium supplemented with 10% fetal bovine serum, 1% penicillin/streptomycin, and 1% glutamine (Gibco). The human TNBC cell line MDA-MB-231 was purchased from ATCC, and the mouse TNBC cell line C0321 was generated from Lfng-/- mouse on FVB background as described (93). TNBC PDX cell lines, 2K1 and 4IC, were generated as described (94, 95). The cDNA encoding Notch1-intracellular domain (Notch1-IC) was subcloned into pBABE-puro vector (Cell BIOLABS, INC). Notch1-IC was transfected into MDA-MB-231 cells using Lipofectamine 2000. Stable Notch1-IC expressing and vector control cells were selected under puromycin and Notch1 expression was confirmed by Western blot analysis. For *ex-vivo* tumor-spheroid/organoids experiments, mouse C0321 cells were transformed with a pmCherry-N1 cloning vector (Life Science Market) using Lipofectamine 2000. The stable mCherry expressing C0321 cells were selected under kanamycin and then enriched with flow cytometry sorting (BD FACSAria II cell sorter, BD Biosciences).

### γ-secretase modulator activity

Plasmid encoding APP CTF (APP C99) and Notch1 juxtamembrane region (NOTCH1) were constructed as reported (39). HEK 293T PS1 (PS1+/+, PS2-/-) and PS2 (PS1-/-, PS2+/+) cell lines were established as described in (96). APP C99 and NOTCH1 were transiently transfected into HEK 293T wild type, PS1, and PS2 cells using polyethyleneimine. After 16 h of incubation, fresh media with different concentrations of SS or SF were added. Conditioned media were collected after 24 h and assayed by Aβ ELISA as described (39). H4 cells stably overexpress APP C99 and NOTCH1 were treated with different concentrations of SS. Conditioned media were collected after 24 hours and assayed by Aβ ELISA (39).

### Mammosphere culture

Primary mammospheres were obtained as previously explained (14). Briefly, MDA-MB-231, C0321, or PDX cells were cultured in MammoCult Human Medium (STEMCELL Technologies) in ultra-low attachment 6-well plates (Corning). After seven days, the primary mammospheres were dissociated into single cells using trypsin and replated in the presence of different concentrations of SS (5, 10, 25, 50, or 100 μM) alone or in combination with 5 μM MK-2206 (AKT-inhibitor). After one week of treatment, mammospheres with a diameter of >100 μm were counted using a Nikon Eclipse microscope. Results were represented as the percentage of mammospheres where control mammospheres were 100%.

### Western blot analysis

Western blot analysis was performed as previously described (14). Briefly, cells were lysed in RIPA buffer (Santa Cruz Biotechnology) and 1 mM Protease and Phosphatase Inhibitor Cocktail (ThermoScientific). The protein samples were resolved in 7.5% Criterion TGX Precast Gels (Bio-Rad), transferred to PVDF membranes (Immobilon-FL Transfer Membrane, Millipore), and blocked in Odyssey blocking buffer (LI-COR). Membranes were incubated with primary antibodies against Notch1 (D1E11), Notch1-IC (Val1744:D3B8) (Cell Signaling), GAPDH, and β-tubulin (Santa Cruz Biotechnology). Following incubation with secondary antibodies (goat anti-mouse 680RD or goat anti-rabbit 800CW; LI-COR), the results were analyzed using the LI-COR Odyssey imaging system.

### T-cell proliferation assay

T-cell proliferation was measured using CFSE dye dilution flow cytometric measurement as described (97). Briefly, T-cells were enriched from naïve FVB mouse spleen using a T-cell (CD3) isolation kit (Stemcells Technologies). Isolated T-cells were then labeled with 1 μM CFSE and plated in a 24-well culture plate with plate-bound anti-CD3 and anti-CD28 (1 μg/ml each). T-cells were treated with SS (5, 25, or 50 μM) at the beginning of incubation, and T-cell proliferation was measured after 72 h by CFSE dilution using flow cytometry. Bone marrow-derived MDSCs (BM-MDSCs) were generated from FVB mice as described (98). Briefly, bone marrow cells were harvested from FVB mouse femur and tibia bones and cultured with G-CSF, GM-CSF, and IL-6 (20 ng/ml each) for four days to generate BM-MDSCs in the presence or absence of SS (5, 25, or 50 μM). CFSE labeled T-cells were co-cultured with BM-MDSCs at a 4:1 (T-cells: MDSC) ratio with SS (5, 25 or 50 μM) in a 24-well culture plate with plate-bound anti-CD3 and anti-CD28 (1 μg/ml each). T-cells proliferation was measured after 72 h by CFSE dilution using flow cytometry.

### Organoid culture

Organoids were derived from syngeneic TNBC C0321 tumors as described (94) with a slight modification. Tumors were harvested, minced, and digested at 37^°C^C in DMEM/F12 Glutamax complete medium (10% FBS, 1% penicillin/streptomycin; Gibco) containing 1mg/ml type IV collagenase (Gibco). The digested tumor was passed over a 70 μm and a 40 μm strainer to isolate organoids of 40-70 μm. The organoids were resuspended in type I rat tail collagen and plated in 8-well chambers (Nunc™ Lab-Tek™ II Chamber Slide™, ThermoScientific). The organoids-collagen cultures were incubated at 37°CC for 30 min to allow the collagen to solidify. The cultures were then hydrated with DMEM/F12 Glutamax complete medium. The organoids were treated with 1 or 5 μM SS with or without 1 μg/ml α-PD1/α-PDL1. Live cells were stained using CellTracker™ Red CMTPX (Invitrogen), and dead cells were labeled using a cell membrane-impermeable dye, NucGreen™ Dead 488 ReadyProbes™ (Invitrogen). Organoids were imaged on days 4-6 using a BZ-X800 (Keyence) microscope.

### *In vivo* experiments

All animal studies were approved by the Institutional Animal Care and Use Committee (IACUC) at the Louisiana State University Health Sciences Center (LSUHSC). Tumors were induced by injecting 1 million TNBC C0321 cells into syngeneic mice with 1:1 ratio of Matrigel to PBS into the mammary fat pad of 6-10-weeks old female FVB mice (Jackson Laboratory). Upon detection of a palpable mass, mice were treated with SS alone (60 mg/kg by PO) daily for another 14 days. For combination immunotherapy experiments, palpable tumors were treated with SS (20 mg/kg, daily, PO) alone or in combination with α-PD1 (100 μg/mouse twice per week) for another two weeks. Tumor volume and body mass were monitored for 21 days from the injection of C0321 cells. Tumors were harvested and were either processed for flow cytometry analysis or formalin fixed and paraffin embedded (FFPE). FFPE tissues were sectioned at 4 microns in thickness and stained with H&E to examine tumor morphology. Immunohistochemistry for Notch1 and Jagged1 was performed as previously described (99); antibodies used included a rabbit polyclonal anti-Notch-1 (Abcam, ab27526, 1:500 dilution), and a rabbit polyclonal raised against aminoacids 1110-1223 of human Jagged1 (Santa Cruz Biotechnology, (H-114, 1: 500 dilution). For flow cytometry analysis, tissues were digested with Liberase and tumor single cells suspensions were analyzed for tumor-infiltrating T-cells (CD4 and CD8), dendritic cells (CD11c), MDSCs (CD11b+Gr1), and TAMs (CD11b+F4/80). All cells were gated on the leukocyte markers (CD45+).

### RNA sequencing

RNA sequencing was done at the Translational Genomics Core (TGC) at the Stanley S. Scott Cancer Center, LSUHSC, New Orleans, LA. RNA was isolated from control, or SS-treated tumor tissues using an RNA isolation kit (Qiagen) per the manufacturer’s protocol. RNA integrity was analyzed on Agilent BioAnalyzer 2100 (Agilent). Paired-end libraries (2 x 75) were prepared using the TruSeq Stranded mRNA Library Prep kit, validated, and normalized following the manufacturer (Illumina, San Diego, CA) protocol. Libraries were sequenced in the NextSeq500 using a High Output Kit v2.5, 150 cycles from Illumina.

### Bioinformatics analysis

Bioinformatics analysis of RNA-Seq data was performed at LSUHSC’s Bioinformatics and Data Science Service Center. We processed the data, raw sequence reads to remove probe IDs with very low and or no expression values across all samples, from the gene expression data matrix. We mapped the probes onto the Ensemble database using BioMart, an Ensemble tool to identify the corresponding gene symbols or names (100). The resulting gene expression data set with gene names was normalized using quantile normalization (101). Using normalized data, we performed supervised analysis comparing gene expression levels between treatment and control samples using a t-test implemented in Pomelo2 (102). This unbiased approach was conducted to identify all genes significantly (p < 0.05) responsive to treatment. In addition to the p-values, we computed the log fold change (Log2(FC)). We used the false discovery rate (FDR) to correct for multiple hypothesis testing (103). The resulting set of genes were ranked based on P-value, FDR and log2(FC). Significantly differentially expressed genes were subjected to unsupervised analysis using hierarchical clustering implemented in the Morpheus software package (104) to determine their patterns of expression profiles. For hierarchical clustering we used the Pearson correlation as the measure of distance between pairs of genes and complete linkage as the clustering method. We performed functional analysis using the Ingenuity Pathway Analysis (IPA) software platform (105) the signaling pathways dysregulated in response to treatment. Under this approach, differentially expressed genes responsive to treatment were mapped onto networks and canonical pathways, using IPA. We used Fisher’s exact *t*-test implemented in IPA to determine the probability of correctly predicting the pathway onto which the gene maps. The pathways were ranked on log-*p*-values and the significant ones were selected. We performed Gene Ontology (GO) analysis implemented in IPA to categorize genes according to the cellular components, molecular functions and biological processes in which they are involved (106).

## Results

### Sulindac sulfide inhibits Notch1 cleavage

**Figure 1:**
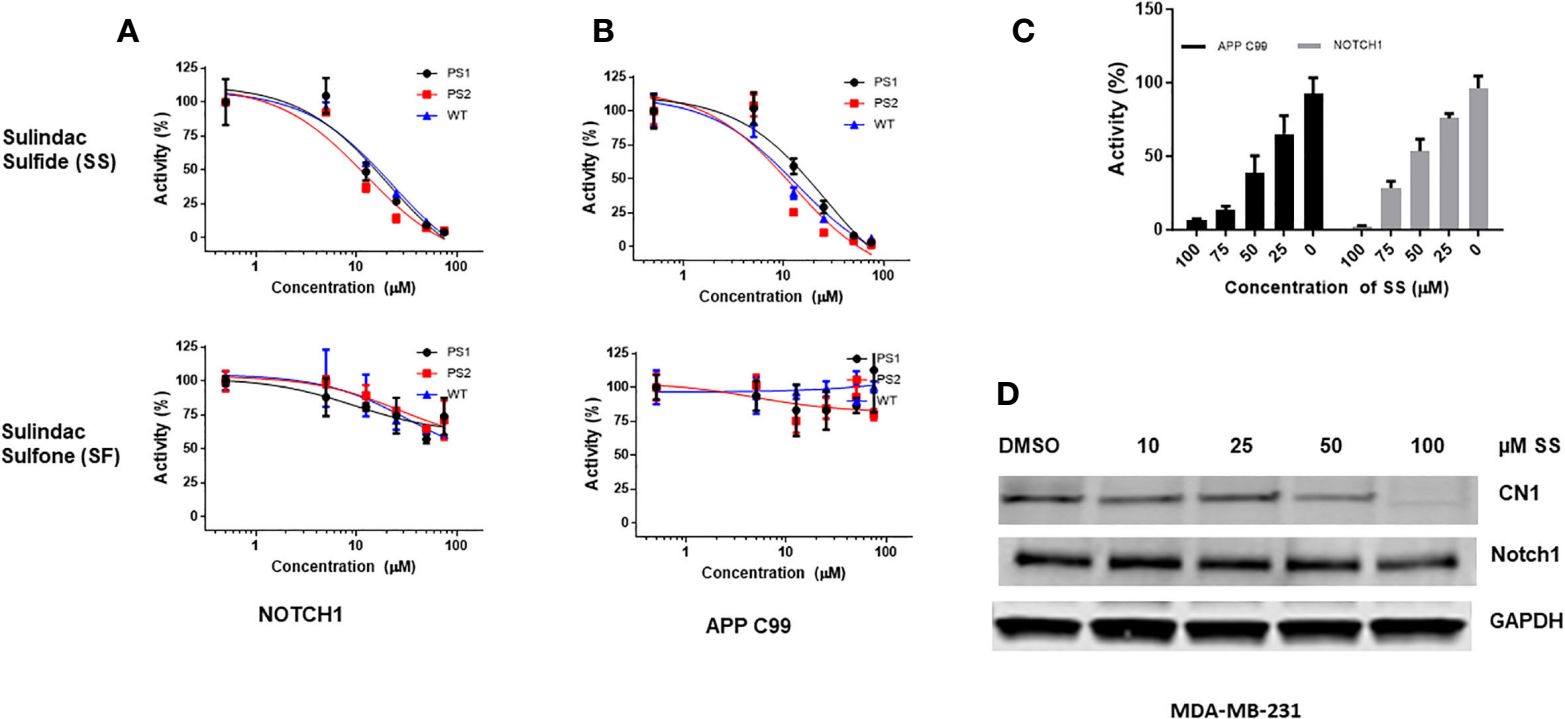
SS has gamma secretase modulator (GSM) activity and inhibits Notch1 cleavage. APP C99 and NOTCH1 were transiently transfected into HEK 293T wild type, PS1, and PS2 KO cells. After 16 hours, cells were treated with 5, 12.5, 25, 50, and 75 μM SS or SF in fresh media. Conditioned media were collected after 24 hours and assayed by Aβ ELISA as described in the method section **(A, B)**. Similarly, H4 cells stably overexpress Notch1 or APP C99 were treated with 25, 50, 75, and 100 μM SS; conditioned media were collected after 24 hours and assayed by Aβ ELISA **(C)**. Human MDA-MB-231 cells were treated with 10, 25, 50, and 100 μM SS for 48 hours, after which the expression of total Notch1 and cleaved Notch1 (CN1) was measured by Western Blot analysis **(D)**.

Previously, the Golde laboratory and others demonstrated that some NSAIDs, including SS, have γ-secretase modulator (GSM) activity (107–110). SS has both GSM and GSI activity at higher concentration (50). Sulindac is a prodrug, which is metabolized into SS and SF by liver enzymes and colonic bacteria (111). SS is a non-selective COX-1 and COX-2 inhibitor and mediates the anti-inflammatory effects of sulindac (64). SF lacks COX-1 and COX-2 inhibitory activity or anti-inflammatory properties, but retains a number of off-target activities (64). We sought to confirm the GSM activity of sulindac derivatives SS and SF in parallel. Therefore, we tested the effects of SS and SF on γ-secretase cleavage of Notch1 and APP-C99 (β-amyloid precursor peptide, a positive control) using HEK wild-type or presenilin-1 (PS1) and presenilin-2 (PS2) KO cells. PS1 and PS2 are catalytic subunits in the γ-secretase complex which are necessary to cleave amyloid precursor protein, generating β-amyloid (112). Therefore, the PS1 and PS2 KO HEK cells were used as controls for γ-secretase activity. SS significantly inhibited Notch1 and APP C99 cleavage, but SF had a very modest effect (**Figures 1A, B**). We therefore focused on SS. Next, we tested SS on H4 cells stably overexpressing APP-C99 and Notch1 to confirm GSM activities. SS inhibited the cleavage of Notch1 and APP-C99 in a dose-dependent manner (**Figure 1C**). Similarly, SS inhibited the γ-secretase mediated release of the intracellular cytoplasmic domains of Notch1 (Notch1-IC in human TNBC MDA-MB-231 cells (**Figure 1D**). Our results suggest that SS has GSM activities and can be tested as a candidate Notch cleavage inhibitor in TNBC.

### SS inhibits mammosphere growth

**Figure 2:**
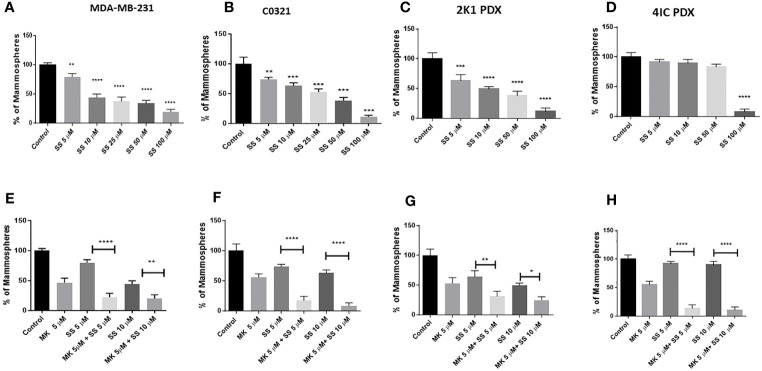
SS inhibits TNBC mammospheres growth in human and murine TNBC models. Mammospheres were grown in Mammocult media (Stemcell Technologies) and P1 mammospheres were then treated with increasing doses [(5, 10, 25, 50, and 100 μM) of SS for one week (twice/week)]. Following incubation, mammospheres were counted using a Nikon microscope and presented as a percentage of control mammospheres; **(A)** human MDA-MB-231, **(B)** mouse C0321 **(C)** human PDX 2K1, and **(D)** human PDX 4IC. In parallel, P1 mammospheres were treated with SS alone (5 or 10 μM) and in combination with AKT inhibitor MK-2206 (MK, 5 μM) for one week (twice/week). Following incubation, mammospheres were counted using a Nikon microscope and presented as a percentage of control mammospheres; **(E)** human MDA-MB-231, **(F)** mouse C0321 **(G)** human PDX 2K1, and **(H)** human PDX 4IC. Data are means ± SD; P-values: *P < 0.05; **P < 0.01; ***P < 0.001; ****P < 0.0001, one-way ANOVA for multiple comparisons, using GraphPad Prism.

The mammosphere formation assay provides an informative and convenient *in vitro* tool to study sphere-forming CSC (113). However, this assay does not address the complexity of CSC formation and maintenance in an *in vivo* niche. Previously, we showed that clinical investigational GSIs are not pharmacologically equivalent, and GSI PF-3084014 (nirogacestat) had the most potent mammosphere inhibitory activity in TNBC cell lines (39). Additionally, we observed an additive effect of GSI PF-3084014 on mammosphere growth in combination with AKT inhibitor MK-2206 (14). Here, we sought to determine whether SS alone or in combination with AKT inhibitor MK-2206 has mammosphere inhibitory activity analogous to GSIs. We tested the activity of SS in three models, 1) human TNBC MDA-MB-231 cells, 2) mouse TNBC C0321 cells, and 3) TNBC PDX cells. SS inhibited mammosphere growth in both MDA-MB-231 and mouse C0321 cells in a dose-dependent manner (**Figures 2A, B**). As previously observed with GSI PF-3084014, when we combined SS with MK-2206, we observed an additive effect on mammosphere growth in both cell lines (**Figures 2E, F**).

PDXs are important models to test human cancer experimental therapeutics (114). Recently, in collaboration with the Burow lab, we characterized several different PDX models from TNBC patients (95, 115, 116). Cell lines were generated from PDX tissues and plated as monolayers as described (14). We developed mammospheres from those cell lines using MammoCult media as described in the Methods section and then tested the efficacy of SS to inhibit mammosphere growth. We found that SS dose-dependently decreased mammosphere growth in 2K1 PDX (**Figure 2C**) but not in 4IC PDX (**Figure 2D**). This PDX model derived from a highly aggressive TNBC that was multi-drug resistant and ultimately fatal (117). Interestingly, when we combined SS with AKT inhibitor MK-2206 we observed an additive effect on mammosphere growth from both PDX models (**Figures 2G, H**). Overall, these results reveal that SS has single agent anti-mammosphere activity in different TNBC models and enhances the activity of an AKT inhibitor in a multi-drug resistant PDX model. This is consistent with the findings of Bhola et al. (30) who reported that inhibition of the PI3K-mTOR-AKT pathway in TNBC increases Notch1 expression in TNBC, and Notch inhibition restores sensitivity to inhibitors of this pathway.

**Figure 3:**
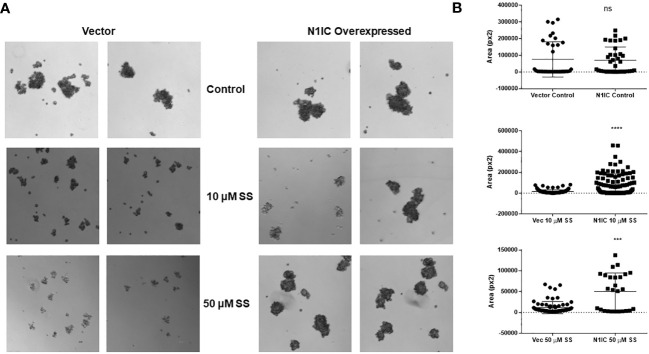
SS-mediated anti-mammosphere activity depends on Notch expression. Vector control and intracellular Notch1-overexpressing (N1IC) MDA-MB-231 cells (10,000) were grown in Mammocult media and treated with SS (10 or 50 μM) for one week (twice/week). Following incubation, mammospheres were counted using a Nikon microscope. Representative photographs and average mammospheres sizes (areas) are presented in **(A, B)** respectively. Data are means ± SD; P-values: ***P < 0.001; ****P < 0.0001, student t-test, using GraphPad Prism. ns, not significant.

Next, we explored whether the anti-mammosphere activity of SS depends on Notch signaling. To answer this question, we generated stable MDA-MB-231 cells expressing cleaved, intracellular Notch1 (Notch1-IC) as described in the Methods section. Overexpression of Notch1-IC was confirmed by Western blotting (**Supplementary Figure 1**). We developed mammospheres from control (vector-transfected) and Notch1-IC overexpressing cells and treated them with SS (5 or 50 µM) or vehicle. Notch1-IC overexpressing cells developed larger mammospheres compared to controls (**Figure 3**) and were essentially insensitive to SS treatment (**Figure 3**). These results indicate that overexpression of active Notch1 rescues the anti-mammosphere activity of SS. As an additional control, we tested whether a selective COX-2 inhibitor, rofecoxib, had anti-mammosphere activity in our hands. Our results showed no effect of rofecoxib over a wide range of concentrations (**Supplementary Figure 2**). These results support the conclusion that the anti-mammosphere activity of SS is unrelated to COX inhibition.

### Characterization of a syngeneic TNBC mouse model: C0321 tumor-infiltrating immune cells

We characterized the tumor microenvironment of a Notch-driven, immune-competent murine transplantable TNBC model developed by the Xu lab from targeted, conditional knockout of Lunatic Fringe (*LFng-/-*) in mice of FVB background (93). LFng-deficient tumors and cell lines expressed high levels of Notch ligand Jagged1 protein and mRNA levels and showed constitutive Notch activation (93). These tumors recapitulate the molecular profiles of human mesenchymal (claudin-low) and basal-like TNBCs (118). Two transplantable clones were isolated from these tumors (93), a mesenchymal/claudin-low clone (C0321) and a basal-like clone (B5725). Here, we used C0321 cells to develop an immunocompetent, Notch-driven TNBC mouse model by injecting 1 million cells into the mammary fat pad of syngeneic FVB female mice. We characterized tumor growth kinetics and tumor-infiltrating immune cell populations in the model. Three weeks after tumor injection, we harvested tumors to generate single cells suspension for flow cytometric analysis of tumor-infiltrating immune cell populations. We found tumor-infiltrating T-cells (CD4, CD8), TAMs, MDSCs, and immune checkpoints including PD1, Lag3, and CTLA4 in C0321 tumors (**Supplementary Figure 3**). These findings support the use of this TNBC syngeneic mouse model for *in vivo* experiments including combination immunotherapy with checkpoint inhibitors.

### SS delays TNBC tumor growth and alters tumor-infiltrating immune cells

**Figure 4:**
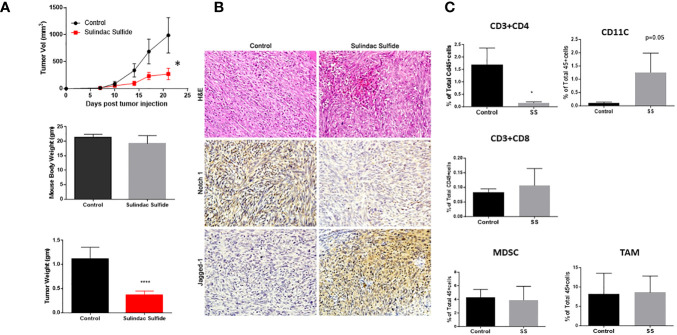
SS monotherapy inhibits the growth of a syngeneic TNBC model (C0321). Mouse TNBC C0321 cells (1 million) were injected into the mammary fat pads of syngeneic immunocompetent FVB (female) mice with 1:1 ratio of Matrigel. Palpable tumors were treated with vehicle or SS (60mg/kg, daily, PO) for another two weeks. Tumor volumes and weights were measured twice per week, and three weeks after tumor inoculation, tumors were harvested, weighed, and analyzed by H&E and immunohistochemistry for Notch1 and Jagged1 **(A, B)**. Fresh tumor specimens were dissociated by Liberase digestion, and single-cell suspensions were analyzed for tumor-infiltrating T-Cells (CD4 and CD8), Dendritic cells (CD11c), MDSC, and TAM **(C)** by Flow cytometer. All cells were gated on pan-leukocyte marker CD45. Data are means ± SD; P-values: *P < 0.05; ****P < 0.0001, Student t-test, using GraphPad Prism.

Several NSAIDs, including sulindac, have received considerable attention as potential chemopreventive agents, as reviewed in (59). In combination with epirubicin, sulindac showed preliminary anti-tumor activity in phase I clinical trials in patients with advanced malignancies, including breast cancer, thus encouraging further investigation (119). Sulindac and docetaxel were tested in a phase II clinical trial in recurrent or metastatic breast cancer (NCT00039520). Yin et al. reported that as single agent sulindac was effective against 4T1 murine breast cancer models and increased the survival of tumor-bearing mice (71). These authors reported that, in their model, sulindac was ineffective in nude mice, suggesting the importance of immune cell-mediated anti-tumor effects of sulindac. Therefore, we wanted to test the efficacy of SS as a single agent using our immunocompetent mouse model (which differs in genetic background from 4T1). We injected C0321 cells into the mammary fat pads of syngeneic FVB female mice. Upon detection of palpable tumors, mice were treated with SS or vehicle for 14 days. SS significantly delayed tumor growth and reduced tumor mass without altering body mass (**Figure 4A**). H&E histopathology revealed increased leukocyte infiltration within the tumor microenvironment upon treatment with SS (**Figure 4B**). SS treatment abrogated Notch1 protein expression in the tumors, but increased Jagged1 expression (**Figure 4B**). Of note, Jagged1 is also a γ-secretase substrate, and recent observations indicate that its cleaved C-terminal fragment has oncogenic activity mediated by a transcriptional complex containing DDX17, SMAD3, and TGIF (120). Whether inhibition of Jagged1 cleavage contributes to the anti-neoplastic activity of SS in this model deserves further investigation.

Next, we analyzed tumor-infiltrating immune cells by flow cytometry (**Figure 4C**). SS did not affect the percentage of tumor-infiltrating MDSCs or TAMs. SS significantly reduced the number of CD4 T-cells and, although not statistically significant, increased the number of CD8 T-cells upon SS treatment. In the BALB(c)-derived 4T1 model, sulindac caused a significant increase in infiltrating CD8 T-cells, which were required for anti-tumor activity (71). In our model, the increase in CD8 T-cells was not statistically significant, while the most remarkable effect was a significant increase in the number of antigen-presenting CD11c+ DC. These results suggest that SS may be investigated in combination with immunotherapy.

**Figure 5:**
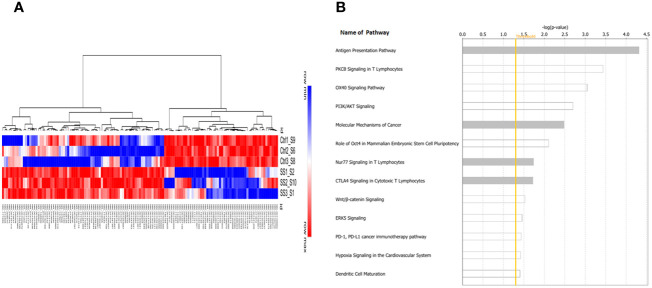
Tumor Gene expression profiling and pathway analysis. The whole transcriptome RNA sequencing of tumor samples was performed in the Translational Genomics Core (TGC) at the Stanley S. Scott Cancer Center, LSUHSC. Bioinformatic analysis was performed as described in the Methods section. A heat map of the 131 genes showing significant modulation by SS versus control **(A)** and a bar graph of the most significantly affected pathways as determined by Ingenuity Pathway Analysis (IPA) **(B)** are shown. The yellow line indicates the threshold level above which the pathway is predicted to be significant using IPA. Note the modulation of antigen presentation pathways as well as multiple immunologically relevant pathways (e.g. PKCθ, OX40, CTLA4, PDL1/PD1, Nur77, DC maturation) as well as pathways relevant to CSC maintenance (Molecular Mechanisms of Cancer, Hypoxia, PI3K/AKT, Wnt, ERK5), all of which cross-talk with Notch.

Next, we performed whole transcriptome RNA-sequencing of control and SS-treated C0321 tumors. We identified 131 differentially expressed genes in SS treated tumors compared to control tumors that passed our FDR and significance thresholds (**Figure 5A**). IPA analysis revealed the most significant pathways following SS treatment (**Figure 5B**). Notably, the most significantly affected pathway was Antigen Presentation, supporting our flow cytometric results. Additionally, several other immunologically relevant pathways were modulated by SS (PKCθ, OX40, CTLA4, PDL1/PD1, Nur77, DC maturation) as well as pathways relevant to CSC maintenance (Molecular Mechanisms of Cancer, Hypoxia, PI3K/AKT, Wnt, ERK5), all of which cross-talk with Notch. Overall our results are consistent with the hypothesis that SS has a multi-targeted anti-tumor effect in this model, including Notch inhibition, inhibition of CSCs and immune-stimulatory effects.

### SS does not suppress T-cell proliferation and blocks BM-MDSC mediated immune-suppressive activity

**Figure 6:**
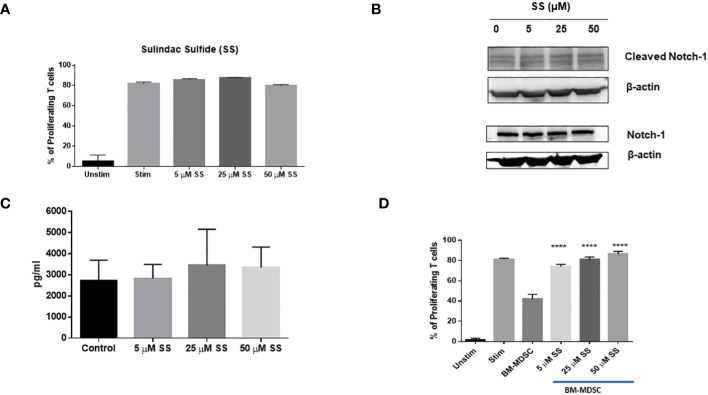
SS does not suppress T-cell proliferation, but blocks BM-MDSC-mediated immune-suppressive activity. T-cells (CD3^+^) were isolated from naïve FVB (female) mice using a negative T cells isolation kit (Stemcell Technologies). Isolated T cells were then labeled with 1 μM CFSE and plated on 24-well culture plates coated with α-CD3 and α-CD28 (1 μg/ml each). T cells were treated with SS (5, 25 or 50 μM SS) at the beginning of incubation, and T-cells proliferation was measured after 72 hours by CFSE dilution using Flow Cytometry **(A)**. Isolated T-cells from naïve FVB (female) were cultured with plate-bound anti-CD3 and anti-CD28 and were treated with SS (5, 25 or 50 μM SS). Following 72 hours of culture, the expression of Cleaved Notch1 (CN1) and Notch1 was measured by Western Blotting **(B)**, and IL-2 production was assessed by ELISA **(C)**. Bone marrow cells were harvested from FVB mice and cultured with GCSF, GM-CSG, and IL-6 (20 ng/ml each) for four days to generate bone marrow-derived MDSC (BM-MDSC) in the presence or absence of SS (5, 25 or 50 μM). CFSE labeled T-cells were co-culture with BM-MDSC at a 4:1 (T-cells: MDSC ratio) with SS (5, 25 or 50 μM) on plate-bound anti-CD3 and anti-CD28 (1 μg/ml each) plate. T-cell proliferation was measured after 72 hours by CFSE dilution using Flow Cytometry **(D)**. Data are means ± SD; P-values: ****P < 0.0001, one-way ANOVA for multiple comparisons, using GraphPad Prism.

Notch inhibitors, including GSIs, are effective in preclinical models of TNBC, where they eliminate CSC resistance to chemotherapy (32, 33, 35, 39–42). However, this strategy has limitations when considering immunotherapy, due to the requirement for Notch signaling in T-cell activation, including CD8 effector T-cells that participate in anti-tumor responses (43–46). Active Notch1 expression renders CD8 T-cells highly resistant to MDSCs and increases their anti-tumor activity (73). Thus, systemic suppression of Notch signaling in TNBC is potentially a double-edged sword; it may successfully target CSCs but may also impair anti-tumor immunity. Therefore, we wanted to determine whether SS affects Notch signaling in T-cells or T-cell proliferation. We isolated T-cells from FVB mouse spleens and performed a T-cell proliferation assay in the presence of increasing concentrations of SS. We found that SS did not significantly affect T-cell proliferation (**Figure 6A**). Consistent with this result, SS did not affect Notch1 cleavage in T-cells at concentrations up to 50 μM, unlike TNBC cells (**Figure 6B**). We also found that SS treatment did not alter IL-2 secretion level in T-cells (**Figure 6C**). Interleukin 2 (IL-2), mainly produced by activated T-cells, plays a central role in controlling the immune response (121). Notch1, activated by APCs carrying Notch ligand DLL4, promotes IL-2 secretion in native CD4 T-cells (122). Altogether, these results indicate that SS at the concentrations tested has no T-cell suppressive activity.

MDSCs promote tumor growth by suppressing cytotoxic T-cell functions (123). The number of MDSCs in the tumor microenvironment was not significantly affected by SS. However, there is evidence that COX inhibition (124) and Notch inhibition by an anti-Jagged monoclonal antibody (73) inhibit the T-cell suppressive activity of MDSCs. Thus, we asked whether SS could inhibit the suppressive functions of MDSCs. We generated bone marrow-derived MDSCs (BM-MDSCs) from FVB mice in the presence of GM-CSF, G-CSF, and IL-6 as described in the Methods section. BM-MDSCs were generated in the presence or absence of SS for four days. We performed T-cell proliferation assays following co-culture with MDSCs treated or untreated with SS. We found that SS significantly blocked the suppressive functions of BM-MDSCs. Importantly, when we added SS during T-cell co-culture with BM-MDSCs, in addition to MDSC maturation, we found significant MDSC activity inhibition even at 5 µM SS concentration (**Figure 6D**). These results suggest a novel anti-immunosuppressive function of SS against MDSCs, which will be further explored in future studies.

### SS enhances the effectiveness of α-PD1 immunotherapy in C0321 organoids

**Figure 7:**
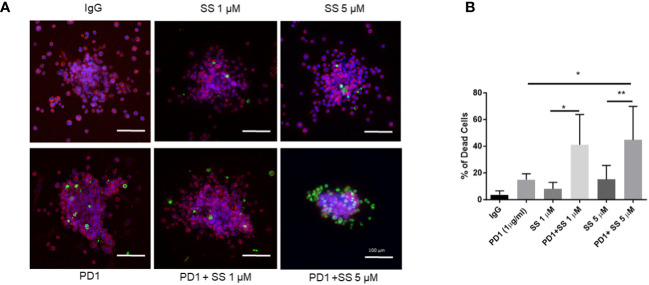
SS enhances the effectiveness of α-PD1 immunotherapy in C0321 organoids. A C0321 tumor from FVB mouse was harvested, minced, and digested to generate organoids as described in the Methods section. Organoids were treated with 1 or 5 μM SS with or without 1 μg/ml of α-PD1. Live cells were stained using CellTracker^™^ Red CMTPX (Invitrogen) and dead cells were labelled using a cell membrane-impermeable dye, NucGreen^™^ Dead 488 ReadyProbes^™^ (Invitrogen). Organoids were imaged using a BZ-x800 (Keyence) microscope **(A)** and the percentage of dead cells was counted using the BZ analyzer software **(B)**. Results represent an average of at least 10 organoids per sample, 3 independent experiments. Data are means ± SD; P-values: *P < 0.05; **P < 0.01, one-way ANOVA for multiple comparisons, using GraphPad Prism.

Tumor organoids recapitulate tumor heterogeneity and microenvironment *in vitro* to enable the of study tumor biology and drug testing (125). The co-existence of tumor cells and immune cells in an intact architecture in tumor organoids makes them suitable three-dimensional tumor culture models (125). We generated organoids from C0321 tumors grown in syngeneic FVB mice as described (126). SS, but not SF, used as a single agent significantly increased tumor cell death in C0321 organoids (**Supplementary Figure 4**). To trace tumor cells within organoids, we developed mCherry-expressing C0321 tumor cells (C0321-mCherry). We generated C0321-mCherry organoids from tumors formed from C0321-mCherry cells following the same protocol we used for unlabeled C0321 cells. C0321-mCherry organoids were treated with SS in the presence or absence of α-PD1. In pilot experiments, we confirmed that SS and α-PD1 caused tumor cell death as single agents and in combination (**Supplementary Figure 5**). We then treated C0321 organoids with SS at 2 concentrations (1 and 5 μM) alone and in combination with α-PD1. SS alone induced cell death in a concentration-dependent fashion. Similarly, α-PD1 caused a significant increase in cell death compared to control IgG. However, combinations of SS and α-PD1 had remarkably higher activity than either agent alone (**Figures 7A, B**). These findings provided a rationale for *in vivo* testing of SS in combination with α-PD1 immunotherapy.

### SS enhances the response of C0321 TNBCs to α-PD1 immunotherapy

**Figure 8:**
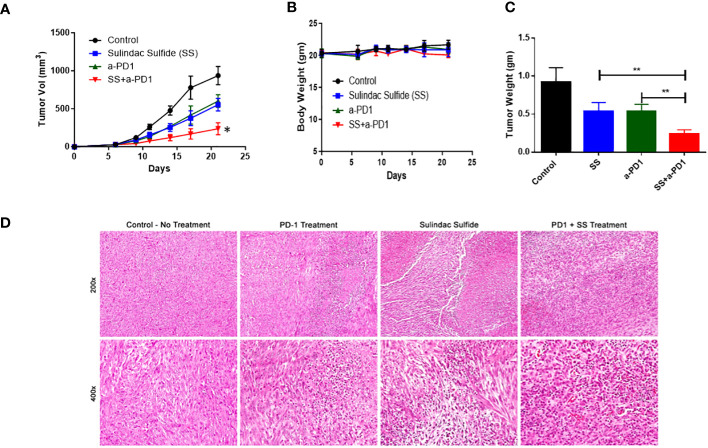
SS enhances the response of C0321 TNBC tumors to α-PD1 immunotherapy. Mouse TNBC cells, C0321 (1 million) were injected into the mammary fat pads of syngeneic immunocompetent FVB female mice in a 1:1 ratio with Matrigel. Palpable tumors were treated with SS (20mg/kg, daily, PO) alone or in combination with α-PD1 (100 μg/mouse twice per week) for another two weeks. Tumor volume **(A)** and weight **(B)** were measured every 3 days. Three weeks after tumor inoculation, tumors were harvested, weighed **(C)**, and stained with H&E **(D)**. Data are means ± SD; P-values: *P < 0.05; **P < 0.01, one-way ANOVA for multiple comparisons, using GraphPad Prism.

Based on our organoid results, we tested the effects of SS at a sub-optimal dose in combination with α-PD1 in the TNBC syngeneic C0321 FVB mouse model. After detection of palpable C0321 tumors, mice were randomized to one of four treatment arms: vehicle control, SS alone (20 mg/kg), α-PD1 or SS plus α-PD1 for another two weeks. We measured tumor growth and mouse body mass during that time. Each single agent showed anti-tumor activity, but the combination of SS and α-PD1 significantly reduced tumor growth compared to either SS or α-PD1 alone (**Figure 8A**). We did not detect significant weight loss or diarrhea during treatment in any of the arms (**Figure 8B**). At the end of the experiment, we harvested tumors. We found that the combination of SS and α-PD1 significantly reduced tumor mass compared to SS or α-PD1 alone (**Figure 8C**). H&E staining (**Figure 8D**) revealed that α-PD1 induced necrosis within tumors. Tumors treated with SS had much larger and extensive areas of necrosis. Combination-treated tumors showed increased inflammatory infiltrates. These results show that SS enhances the response of C0321 TNBC tumors to α-PD1 immunotherapy.

## Discussion

TNBC patients have a high risk of recurrence and metastasis, and current treatment options remain limited (127). The treatment of TNBC poses many challenges, including: 1) Molecular heterogeneity among patients (128–132), with multiple molecular subtypes that lack a common, druggable target; 2) Intra-tumoral heterogeneity, with frequent appearance of multiple chemo-resistant subclones during treatment, resulting in the selection of highly chemo-resistant recurrent tumors (133–135). These clones contain cells with stem-like properties (CSCs) that can cause recurrent disease after remission and rely on redundant signaling pathways (41, 136); 3) Phenotypic plasticity, whereby signals from the microenvironment can reprogram “bulk” cancer cells to acquire a CSC phenotype through epithelial-mesenchymal transition (EMT) (137–139) and 4) Failure of the immune system to eliminate malignant clones (140, 141), due to systemic immune suppression in tumor-bearing patients and immunosuppressive tumor microenvironment, as well as immune editing of the tumor itself, whereby less immunogenic and/or more immune-suppressive clones are selected over time. There is strong evidence for the involvement of Notch signaling in TNBC (13–20). Expression of Notch1 and its ligand Jagged1 correlate with poor prognosis, and expression of Notch1 mRNA correlates with poor survival in recurrent TNBC (21–23). CSC emerging after chemotherapy or targeted agents in TNBC are often Notch-dependent (30–38). Notch inhibitors, including γ-secretase inhibitors (GSI), are quite effective in preclinical models of TNBC, where they eliminate CSC resistance to chemotherapy (32, 33, 35, 39–42). However, GSIs have two pharmacologic liabilities: their well-documented intestinal toxicity, which generally precludes continuous administration, and the potential to suppress tumor immunity. Notch signaling is required for T-cell activation, including CD8 effector cells that participate in tumor responses (47–49). To overcome this impasse, we explored the space of FDA-approved drugs with γ-secretase modulator (GSM) activity and established safety records. Most FDA-approved drugs, particularly older ones, have primary mechanisms of action and several low to intermediate potency off-target effects, which may contribute to their safety and efficacy. We identified sulindac sulfide (SS), the active metabolite of FDA approved NSAID sulindac, as a potential candidate, for the following reasons: GSM activity (90, 107, 109, 110, 142, 143), inhibition of IKKα and β phosphorylation and NF-κB activity (84, 85, 144), and inhibition of COX-1 and -2 enzymatic activity. As a result of COX inhibition, SS prevents the production of PGE_2_, a highly immune-suppressive inflammatory mediator well-known to dampen T-cell responses, including CD8 cytotoxic T-cell activity (70, 75).

Tumor cells and tumor-associated immune cells such as TAMs and Tregs produce PGE_2_. PGE_2_ has multiple pro-tumor immune modulatory effects in the tumor microenvironment such as reduced DC and Th1 T-cell functions and enhanced Treg MDSC and M2 TAM activity [54-58]. Furthermore, SS inhibits Wnt signaling via inhibition of cGMP phosphodiesterase 5, thereby potentially denying an avenue of resistance to simple Notch inhibition (145). SS is the active metabolite of sulindac, an FDA-approved, well-tolerated agent that has been widely studied for its chemopreventive properties (71). A combination of sulindac and epirubicin has been tested in patients with advanced cancer (119). Sulindac sulfone (SF) lacks COX inhibitory activity but retains some anti-tumor properties (146–150). SF has been tested with capecitabine in metastatic breast cancer (151). Here, we compared the GSM activities of SS and SF. We found that SS has significant GSM activity, while SF has very modest GSM activity. Using human and mouse TNBC cells and PDX mammospheres, we demonstrated an anti-mammosphere activity of SS at clinically achievable concentrations. This activity was rescued by Notch1-IC expression, consistent with the notion that GSM blockade of Notch cleavage is at least a major mechanism of action of SS in these experiments. Importantly, we found that SS at the same concentrations active in cancer cells did not affect Notch1 cleavage or expression in activated T-cells, nor did it inhibit T-cell proliferation or IL-2 secretion. We do not know the mechanism for this apparent selectivity of the GSM activity of SS. One possible explanation is that Notch cleavage in activated T-cells takes place not at the cell surface but in acidified endosomes (152, 153). It is possible that SS does not reach sufficient concentrations to affect Notch cleavage in T-cell endosomes. To test the *in vivo* efficacy of SS, we studied a Notch-driven TNBC syngeneic mouse model (C0321) (93). SS inhibited TNBC tumor growth and altered the profile of tumor-infiltrating immune cells. In our model, SS virtually eliminated expression of Notch1 in tumors. This is likely the result of prolonged inhibition of Notch activity, since Notch1 transcriptionally induces its own expression (154) and the expression of furin, the enzyme required for Notch1 precursor protein processing (155). While we cannot rule out that *in vivo* COX inhibition may contribute to Notch inhibition (72, 82, 83), a selective COX-2 inhibition had no effect on mammosphere growth in our hands. Using the 4T1 murine breast cancer model, Yin et al. reported that the anti-tumor effect of sulindac was mediated by CD8 anti-tumor immunity (71). While the increase in intra-tumoral CD8 T-cells we observed was not statistically significant, our results are consistent with the notion that SS stimulates tumor immunity. The C0321 model and the 4T1 model have different genetic backgrounds (FVB for C0321 and BALB(c) for 4T1) (71, 93). In our model, we report a previously undescribed effect of SS, namely, a large increase in intratumoral CD11c+ DC, which was confirmed by increased expression of genes involved in antigen processing as determined by tumor whole-transcriptome RNA-Seq. GSIs have been shown to increase CD11+ DC in a graft-versus-leukemia model (156), although they also increased T-regs. Similarly, inhibition of Jagged1 and 2 via a monoclonal antibody results in increased CD11c+ cells, suppressed MDSC activity and increased CD8 infiltration in murine solid tumor models (73). Thus, it is possible that GSM activity may, at least in part be responsible for the increase in intra-tumoral CD11c+ cells. Since blockade of Jagged-Notch signaling inhibits the immune suppressive functions of MDSC (73), we also explored whether SS affects MDSC activity.

MDSC promote tumor growth by suppressing cytotoxic T-cells functions (123). We describe here a novel anti-MDSC effect of SS. At concentrations as low as 5 μM, SS virtually abrogated the inhibition of T-cell proliferation caused by bone marrow-derived MDSCs. This may be due to inhibition of Jagged-Notch signaling (see above) but also potentially to COX-2 inhibition, which has been showed to suppress MDSC activity (124). PGE2, through its EP4 receptor, increases MDSC activity (157).

We found that SS had significant anti-neoplastic activity in a Notch-driven syngeneic TNBC model as monotherapy, and that it enhanced the effectiveness of α-PD1 immunotherapy in organoids and *in vivo*, without unexpected toxicity at a 3-times lower dose. After a single administration of 200 mg sulindac in humans, SS reaches an average Cmax of 2.44 μg/ml (approximately 7 μM) with an average effective half-life of 16.4 h (158, 159). SS is highly protein-bound and with repeated administration of sulindac (200 mg bid, the standard dose used in chemoprevention trials) it accumulates, reaching steady-state plasma concentrations that are 1.4 to 1.9 higher than those achieved after single administration (111). Hence, the active concentrations used in our mammosphere and organoid experiments are likely to be clinically achievable. Comparing *in vivo* pharmacokinetics between mice and humans is rarely straightforward, but the doses of SS we used in mice (60 and 20 mg/kg) had no appreciable toxicity and are comparable or significantly lower than doses safely used in mouse chemoprevention studies (160, 161). Importantly, no secretory diarrhea was observed, a dose-limiting adverse effect of GSIs. The reason for this difference is unknown. A possible explanation may involve the simultaneous inhibition of Notch and Wnt (the latter via PDE5), which have opposite effects on intestinal crypt stem cell fate decisions (162).

Here, we describe multiple pharmacological activities of SS in TNBC: 1) A GSM activity leading to Notch inhibition and anti-CSC activity in human and murine models; 2) A novel effect on intra-tumoral CD11c+ APC that may also be due, at least in part, to Notch inhibition and 3) A novel inhibitory effect on MDSCs, which may be due to Notch inhibition and/or to COX inhibition. As CSC cross-talk with MDSC and other immune cell populations (163), these effects are likely to compound *in vivo*. *In vivo* experiments demonstrated that SS in our model has single-agent anti-tumor activity and significantly increases the efficacy of α-PD1 immunotherapy at non-toxic doses. Taken together, our data support the investigation of sulindac repurposing as an anti-cancer agent in TNBC with concurrent anti-CSC and immune-stimulatory properties.

## Data availability statement

The datasets presented in this study can be found in online repositories. The names of the repository/repositories and accession number(s) can be found below: Gene Expression Omnibus (GEO) under accession number GSE241064.

## Ethics statement

The studies involving humans were approved by Tulane University Institutional Review Board. The studies were conducted in accordance with the local legislation and institutional requirements. The human samples used in this study were acquired from primarily isolated as part of your previous study for which ethical approval was obtained. Written informed consent for participation was not required from the participants or the participants’ legal guardians/next of kin in accordance with the national legislation and institutional requirements. The animal study was approved by Louisiana State University Health Sciences Center Institutional Animal Care and Use Committee. The study was conducted in accordance with the local legislation and institutional requirements.

## Author contributions

FH and LM contributed to the conception and design of the study. FH, DU, GM, SM, KL, HL, DW, KX, SS, SH, MM, YX, MB, BC-B, LD, CH, YR, JZ, TG, BO performed experiments, analyzed data and/or provided key materials. FH wrote the first draft of the manuscript. LM supervised the study. All authors contributed to manuscript revision, read, and approved the submitted version.

## Supplementary material

Supplementary Figure 1 Western blot confirmation of Notch1-IC (N1IC) expression in MDA-MB-231 cells. MDA-MB-231 cells were transfected with pcDNA3.1 vector or pcDNA3.1-N1IC plasmid using Lipofectamine 2000. Overexpression of N1IC was confirmed by Western Blot.

Supplementary Figure 2 Rofecoxib does not inhibit TNBC mammospheres growth. Human MDA-MB-231 mammospheres were grown in Mammocult media (Stemcell Technologies) and P1 mammospheres were then treated with increasing doses (0.1, 1, 5, and 10 μM) of Rofecoxib (COX-2 inhibitor) for one week (twice/week). Following incubation, mammospheres were counted and presented as a percentage of control mammospheres. Data are means ± SD.

Supplementary Figure 3 Characterization of a syngeneic TNBC mouse model. Mouse TNBC C0321 cells (1 million) were injected into the mammary fat pads of syngeneic immunocompetent FVB female mice with 1:1 ratio of Matrigel. Three weeks after tumor inoculation, tumors were harvested and dissociated by Liberase digestion. Single-cell suspensions were analyzed for tumor-infiltrating immune cells, CD4, CD8, TAM, MDSC, and immune checkpoint markers PD1, Lag3, and CTLA4 by flow cytometry. All cells were gated on pan-leukocyte marker CD45.

Supplementary Figure 4 Sulindac sulfide but not sulindac sulfone causes dose-dependent cell death in C0321 tumor organoids. A C0321 tumor from an FVB mouse was harvested, minced, and digested to generate organoids as described in the Methods section. C0321 tumor organoids loaded onto a 3D microfluidic device were treated with varying concentrations (5µM, 20µM, and 50µM) of sulindac sulfide or sulindac sulfone. On day 6 of incubation, organoids were treated with Acridine orange/propidium iodide (AO/PI) to identify live and dead cells within the spheroids, and the % live and dead cell area was calculated using NIS-elements software. Data are means ± SD; *P*-values: ***P* < 0.01; ****P* < 0.001; *****P* < 0.0001.

Supplementary Figure 5 SS enhances the effectiveness of α-PD1 immunotherapy in C0321 organoids. We generated C0321-mCherry organoids from tumors formed from C0321-mCherry cells following the same protocol we described earlier. C0321-mCherry organoids were treated with SS (1µM) in the presence or absence of α-PD1 (1µg/ml). Organoids were stained using a cell membrane-impermeable dye, NucGreen™ Dead 488 ReadyProbes™ (Invitrogen). Organoids were imaged as described earlier.
