# Supplementary figures and images for: Sulindac sulfide as a non-immune suppressive γ-secretase modulator to target triple-negative breast cancer
Source: Front Immunol. 2023 Oct 13;14:1244159. doi: 10.3389/fimmu.2023.1244159 (PMC10612326; doi:10.3389/fimmu.2023.1244159)

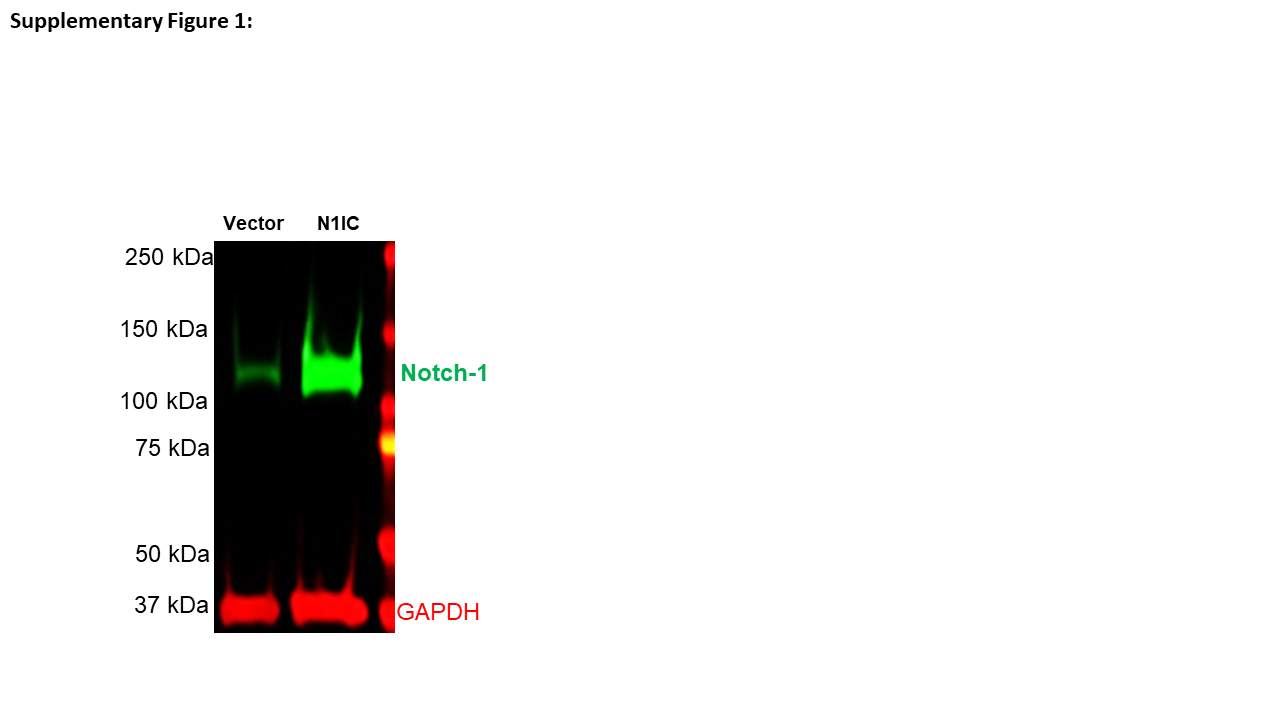

Supplement: Supplementary Figure 1 — Western blot confirmation of Notch1-IC (N1IC) expression in MDA-MB-231 cells. MDA-MB-231 cells were transfected with pcDNA3.1 vector or pcDNA3.1-N1IC plasmid using Lipofectamine 2000. Overexpression of N1IC was confirmed by Western Blot. [file Image_1.tif]

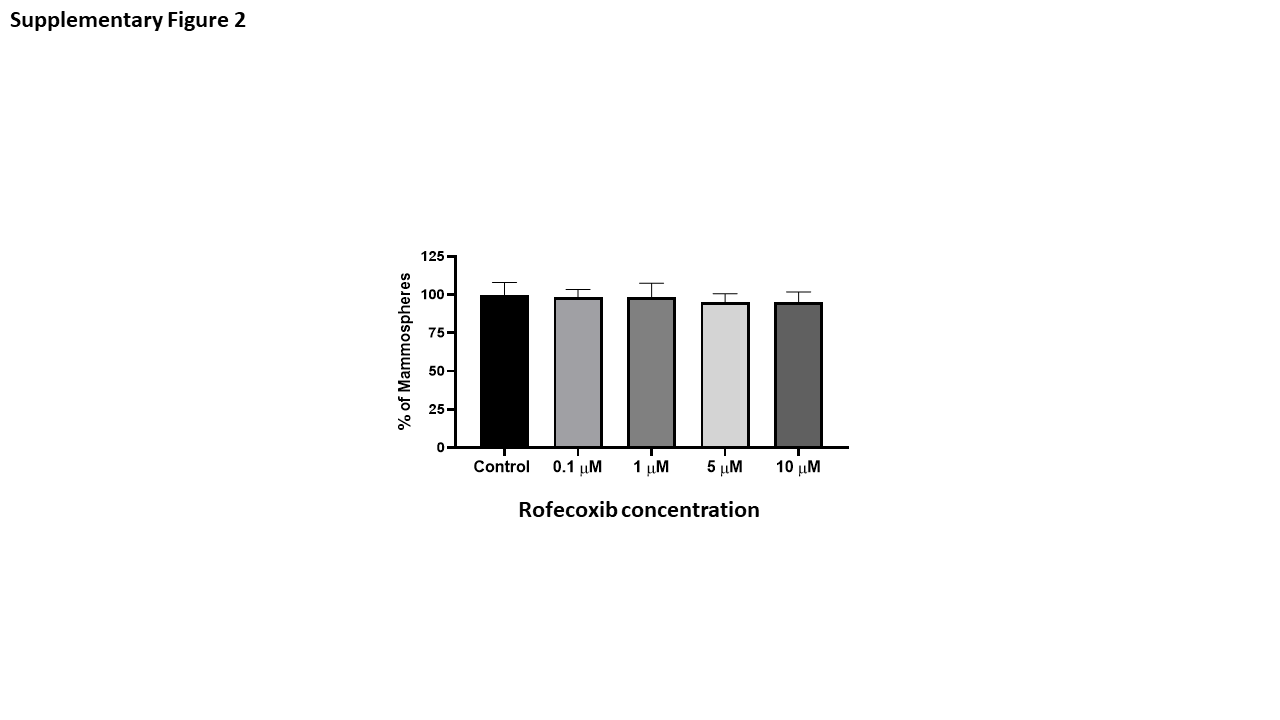

Supplement: Supplementary Figure 2 — Rofecoxib does not inhibit TNBC mammospheres growth. Human MDA-MB-231 mammospheres were grown in Mammocult media (Stemcell Technologies) and P1 mammospheres were then treated with increasing doses (0.1, 1, 5, and 10 μM) of Rofecoxib (COX-2 inhibitor) for one week (twice/week). Following incubation, mammospheres were counted and presented as a percentage of control mammospheres. Data are means ± SD. [file Image_2.tif]

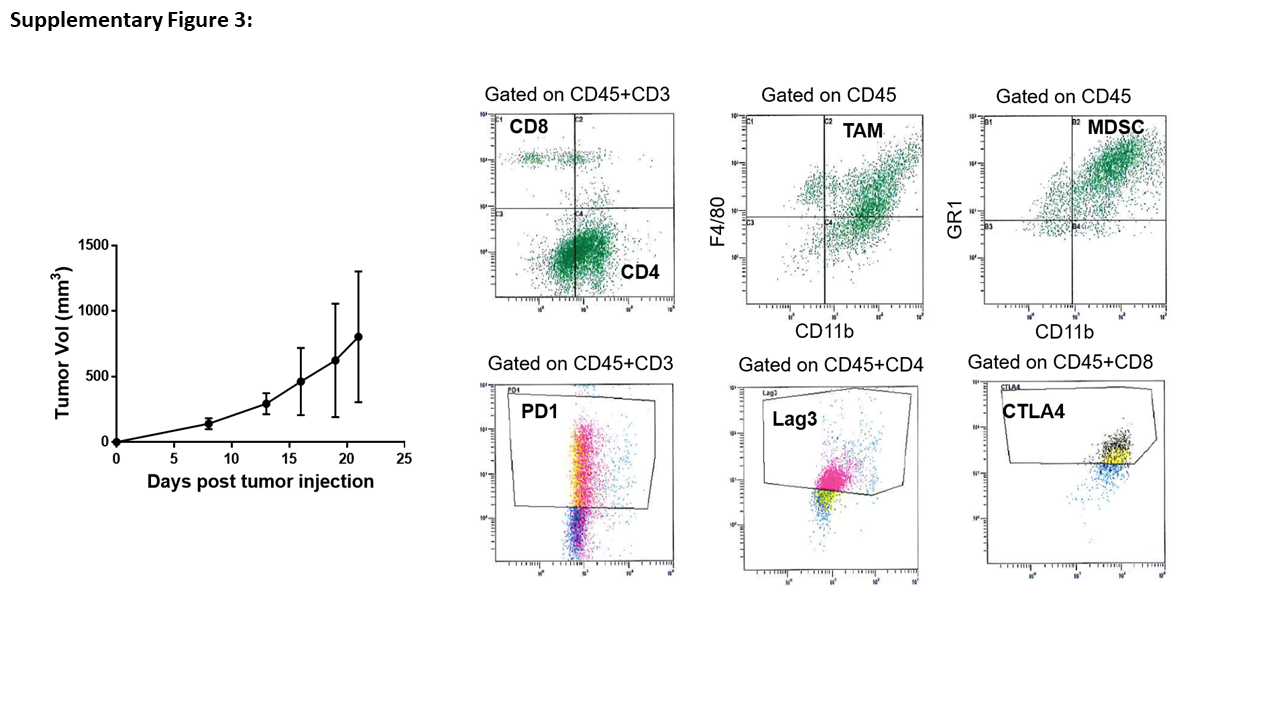

Supplement: Supplementary Figure 3 — Characterization of a syngeneic TNBC mouse model. Mouse TNBC C0321 cells (1 million) were injected into the mammary fat pads of syngeneic immunocompetent FVB female mice with 1:1 ratio of Matrigel. Three weeks after tumor inoculation, tumors were harvested and dissociated by Liberase digestion. Single-cell suspensions were analyzed for tumor-infiltrating immune cells, CD4, CD8, TAM, MDSC, and immune checkpoint markers PD1, Lag3, and CTLA4 by flow cytometry. All cells were gated on pan-leukocyte marker CD45. [file Image_3.tif]

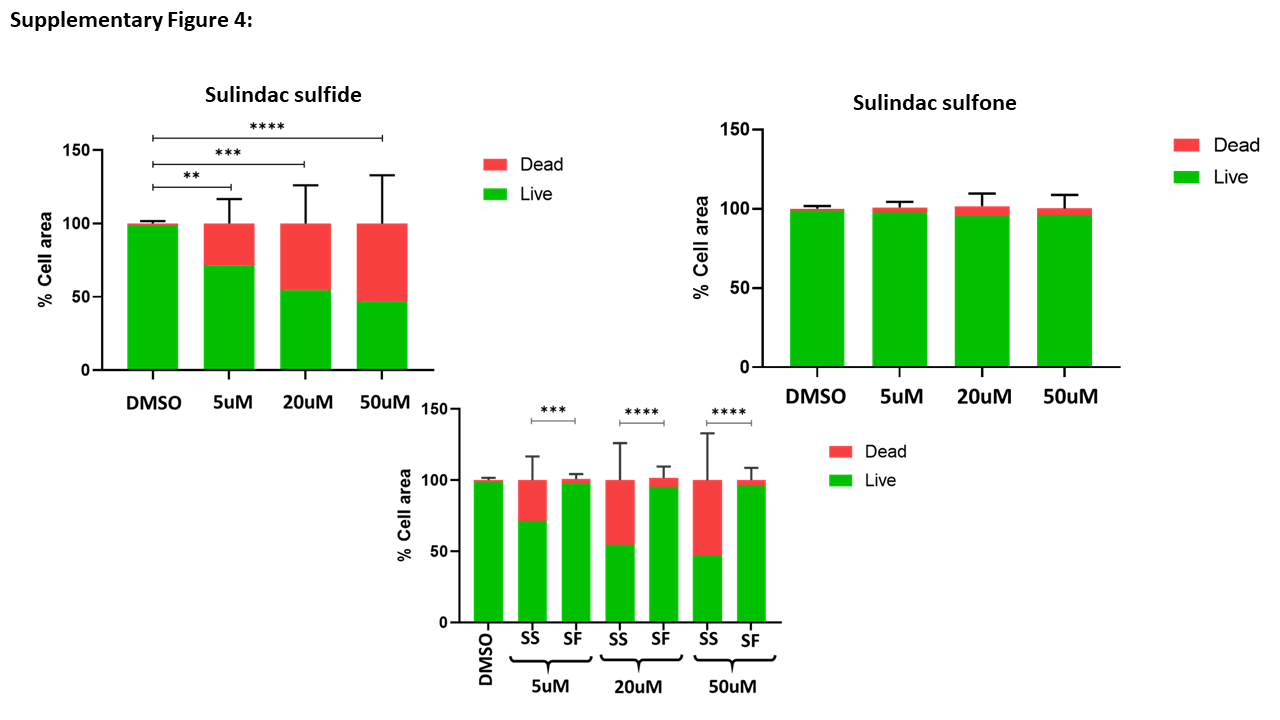

Supplement: Supplementary Figure 4 — Sulindac sulfide but not sulindac sulfone causes dose-dependent cell death in C0321 tumor organoids. A C0321 tumor from an FVB mouse was harvested, minced, and digested to generate organoids as described in the Methods section. C0321 tumor organoids loaded onto a 3D microfluidic device were treated with varying concentrations (5µM, 20µM, and 50µM) of sulindac sulfide or sulindac sulfone. On day 6 of incubation, organoids were treated with Acridine orange/propidium iodide (AO/PI) to identify live and dead cells within the spheroids, and the % live and dead cell area was calculated using NIS-elements software. Data are means ± SD; P-values: **P < 0.01; ***P < 0.001; ****P < 0.0001. [file Image_4.tif]

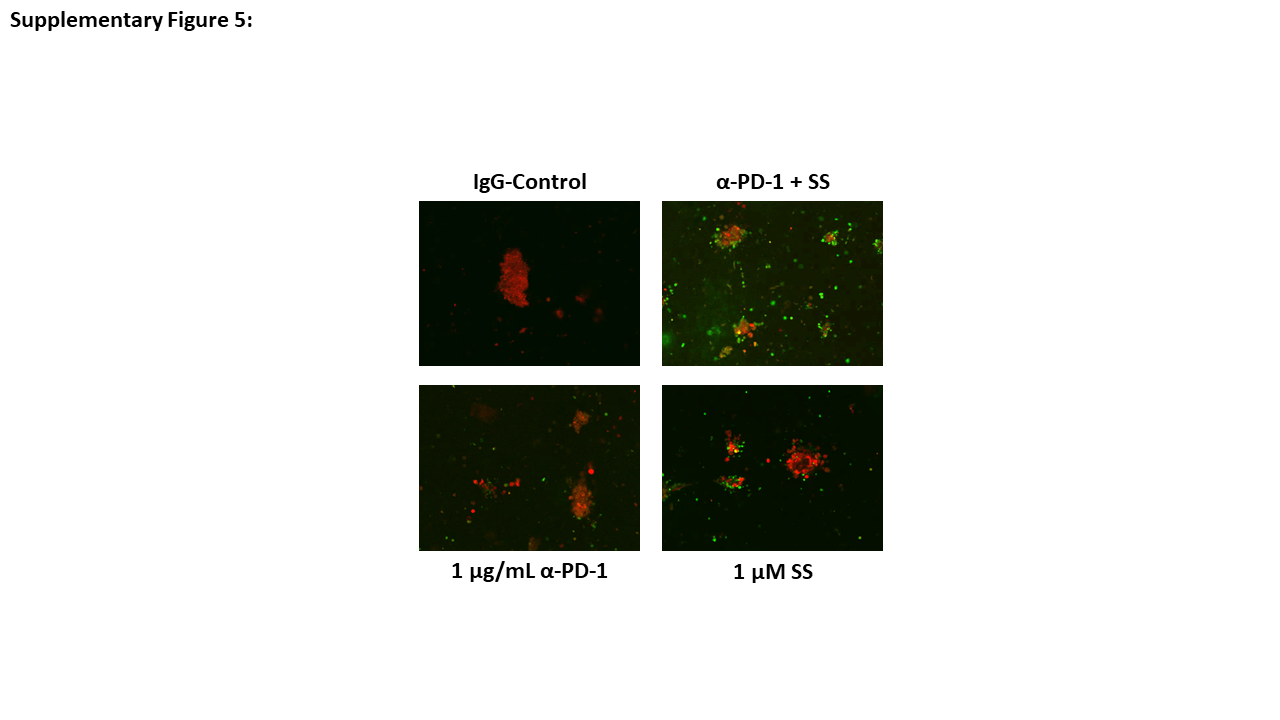

Supplement: Supplementary Figure 5 — SS enhances the effectiveness of α-PD1 immunotherapy in C0321 organoids. We generated C0321-mCherry organoids from tumors formed from C0321-mCherry cells following the same protocol we described earlier. C0321-mCherry organoids were treated with SS (1µM) in the presence or absence of α-PD1 (1µg/ml). Organoids were stained using a cell membrane-impermeable dye, NucGreen™ Dead 488 ReadyProbes™ (Invitrogen). Organoids were imaged as described earlier. [file Image_5.tif]
